# Life in 90 words: opportunities for person-centred care amidst COVID-19

**DOI:** 10.1177/1039856220975280

**Published:** 2020-12-09

**Authors:** Theo Theodoros, Marianne Wyder, Chiara Lombardo, Frances Dark, Anup M Joseph, Anne Steginga, Sharon Locke, Kieran Kinsella, Manaan Kar Ray

**Affiliations:** Addiction and Mental Health Services, Princess Alexandra Hospital, Metro South Hospital and Health Service, Australia; PA-Southside Clinical Unit, Princess Alexandra Hospital, Faculty of Medicine, University of Queensland, Australia; Addiction and Mental Health Services, Princess Alexandra Hospital, Metro South Hospital and Health Service, Australia; Research and Development, Mental Health Foundation, UK; Institute for Health and Human Development, University of East London, UK; Addiction and Mental Health Services, Princess Alexandra Hospital, Metro South Hospital and Health Service, Australia; PA-Southside Clinical Unit, Princess Alexandra Hospital, Faculty of Medicine, University of Queensland, Australia; Addiction and Mental Health Services, Princess Alexandra Hospital, Metro South Hospital and Health Service, Australia; PA-Southside Clinical Unit, Princess Alexandra Hospital, Faculty of Medicine, University of Queensland, Australia; Addiction and Mental Health Services, Princess Alexandra Hospital, Metro South Hospital and Health Service, Australia; Addiction and Mental Health Services, Princess Alexandra Hospital, Metro South Hospital and Health Service, Australia; Addiction and Mental Health Services, Princess Alexandra Hospital, Metro South Hospital and Health Service, Australia; Addiction and Mental Health Services, Princess Alexandra Hospital, Metro South Hospital and Health Service, Australia; PA-Southside Clinical Unit, Princess Alexandra Hospital, Faculty of Medicine, University of Queensland, Australia; and Australian Institute for Suicide Research and Prevention, Griffith University, Australia

**Keywords:** COVID-19, contingency planning, telehealth, care synopses, guided conversations

## Abstract

**Objective:**

Coronavirus disease 2019 and the consequent public health and social distancing measures significantly impacted on service continuity for mental health patients. This article reports on contingency planning initiative in the Australian public sector.

**Methods:**

Ninety-word care synopses were developed for each patient. These formed the basis for guided conversations between case managers and consultant psychiatrists to ensure safe service provision and retain a person-centred focus amidst the threat of major staffing shortfalls.

**Results:**

This process identified vulnerable patient groups with specific communication needs and those most at risk through service contraction. The challenges and opportunities for promoting safety and self-management through proactive telehealth came up repeatedly. The guided conversations also raised awareness of the shared experience between patients and professionals of coronavirus disease 2019.

**Conclusion:**

There is a parallel pandemic of anxiety which creates a unique opportunity to connect at a human level.

In Australia, the first Australian case of novel coronavirus disease 2019 (COVID-19) occurred on the 25th of January 2020. The World Health Organisation declared a pandemic on the 11th of March 2020. In response, governments globally initiated stringent public health measures (including social distancing) to protect lives. Compared globally, case numbers in Australia are extremely small but the public health response has been similar. Mental health services needed to adapt and adopt new practices compliant with social distancing, becoming less reliant on in-person appointments. Service continuity was key to this planning process. When staff are infected or exposed to potentially infected persons, there are mandatory self-isolation requirements, and a single team member contracting COVID-19 could force an entire team into self-isolation. Services planned for a worst case 40%–50% staff reduction, as teams could be taken offline overnight due to exposure.

## Methods

### Process of developing contingency plans

As part of contingency planning, in a large Australian public-funded mental healthcare service, brief synopses on each current patient were written. These were informed by scripted telehealth welfare checks and guided conversations with patients. The focus of these synopses was to establish what was needed for patients to stay safe during the restrictions. These care synopses were completed with 1300 patients during the fortnight from the week starting the 16th of March 2020.

***Care synopses.*** Each case manager wrote a 90-word individual care synopsis for every patient in their care. The synopsis aimed to provide: a sense of the person, their core strengths, risk and safety concerns. They also outlined who was responsible for what and when. The synopses were intended to provide a brief patient overview and to be a starting point for triage. The synopses were intended to allow, in the event a case manager or entire team was taken offline or redeployed, for any healthcare professional to take over the care and address immediate needs. Table 1 provides a sample synopsis.

**Table 1.** Sample of a synopsis (artificially created for explanatory purposes)

Team	Mood	Consultant	Dr Thomas
Consumer name	Jane SMITH	Diagnosis	F60.31 – borderline personality disorder F10.21 – alcohol use disorder
NGO care provider and contact	Sexual assault counsellor (Anne Davis)	CIMHA demographics updated and current	Yes
Key carer name and contact	John Smith (husband) 0123456789		
Mental Health Act status	Voluntary	Depot name and frequency (e.g. monthly, fortnightly)	N/A
Treatment red flags/alerts	Prior serious suicide attempt by means of polypharmacy overdose with alcohol co-ingestion (requiring ICU admission) in 2018 History of self-harm by means of superficial laceration with razor blades		
Phase of care	Functional gain		
Recommended care delivery mode	Telehealth for individual contact and video-conference for skills group	Recommended contact	0400 000 000 Weekly contact with case manager (individual DBT therapist) and weekly skills group

*Note.* ICU = intensive care unit, NGO = non-government organisation, DBT = dialectical behaviour therapist, CIMHA = consumer integrated mental health application (Electronic Patient Records used for Mental Health in Public Services across Queensland, Australia).

Synopsis of current care needs, immediate treatment interventions and responsible person/service (word limit 90 words):

21-year-old female; childhood sexual abuse history; living with supportive husband; arts degree part-time by distance; nil children. Nil family in Brisbane. Caring; loves her dog. Alcohol dependence in remission (support from drug/alcohol services). In DBT Skills Program with Mood team (completed 6 months). Self-harm decreasing. Husband requires ongoing psychoeducation to increase support capacity. Main risks (self-harm/suicide) increase with alcohol intoxication. Not requiring psychotropic medication. Engaging with her sexual assault counsellor and GP. Increasing financial stressors with COVID-19 (decreased hours). Increase contact if relapses with alcohol use or self-harm.

***Welfare check.*** The care synopses were enriched by scripted welfare checks. Over 2500 telehealth calls were made in the second half of March 2020. Every patient, and the next of kin for patients who consented, received a call from their case manager to establish their well-being, call preferences and to make a judgement call on the ability to self-manage and suggested frequency of contact. Table 2 provides details of the topics covered.

**Table 2.** Welfare contact for patients and next of kin

**The 5As of appreciative curiosity: common script for all telehealth contacts** • Answer any questions using the 5A principles outlined below ➢ **Acknowledge** concerns, thoughts and feelings ➢ Provide **assurance** around what has been put in place so far ➢ Increase **awareness** of potential resources and contact numbers for available resources ➢ Advise what **actions** the person/family can take in the case of deterioration – reinforce contact numbers ➢ Express **appreciation** for their understanding and involvement
**Welfare contact for patients** **Purpose**: Ascertain the level of contact that will be needed going forward
Update contact • Is the person contactable on phone? • Do they pick up a call from a restricted number? • Do we need to send a text to forewarn a call is coming? • What is the best time to contact them? • Are all numbers (including next of kin) and consent to contact up to date?
**How are they doing?** Establish whether there has been any change • No – reinforce steps to stay well, service continuity plan, how to seek help • Yes – establish the primary reason why? ➢ Anxiety – acknowledge, assurance, awareness of steps they can take ➢ Isolation – how to stay in touch with their loved ones ➢ High expressed emotions – ways to defuse the situation and maintain distance ➢ Symptoms worsening – what do we need to do? (relapse plan/safety plan/medication review/consultant discussion) ■ Make note of actual mental health sequelae of COVID-19 pandemic in terms of overthinking, emerging unhelpful beliefs, paranoia, etc.
**Essential supplies** • Have you got enough food and drink? • How much medication have you got? How do you get your medication? • Have you got credit on your phone? • Who will you get in touch with if things change? • Reinforce natural circle of support and staying in touch from where they are staying • Reinforce contact numbers within service, both in and out of hours
**Contacting designated next of kin** **Purpose**: To enhance the patient’s ability to manage in the community with the potential reduction in face-to-face support by engaging their natural supports wherever possible
**Gather information** • Confirm the next of kin details • Does the next of kin live locally? • Are they able to be in touch with the consumer regularly? • How often will they be able to check in with the consumer via telephone? • Are they able to support the consumer to access practical needs, that is, shopping, medication, etc. if the consumer is isolated at home?
**Provide information** • Confirm the service plan of care for the individual with NOK ➢ Outline relapse prevention plan/safety plan and the part NOK is expected to play ➢ Outline how medical review/case manager contact will occur • Seek agreement about the level of support the NOK can offer and document it in the care plan

Note: NOK = next of kin.

***Guided conversations.*** Once completed, between 30 March and 1 April 2020, a Consultant Psychiatrist met with each case manager individually to discuss the synopses. These discussions focussed on: strengths, needs, risks (particularly, the risk of being lost to follow-up), early warning signs and safety plans. The schedule and type of contact (in person or telehealth) was also established, as well as when to increase support. The guided conversations also focussed on the principles of care during the pandemic and the ability of the person to self-sustain and self-manage. The psychiatrists then provided detailed feedback regarding individual patient concerns to the hospital directors at a daily midday COVID-19 contingency planning meeting.

#### Data analysis

Feedback from guided conversations was thematically organised into three main areas of concerns. Daily meeting minutes, issues log, Gantt charts, redeployment plans, multi-site team operational plans and the monthly governance action log helped to identify and collate these. The findings were presented to the teams through a governance meeting on the 2nd of April 2020. Opportunities for person-centred responses were identified in teams. Progress was tracked through daily planning meetings.

## Results

### Challenges, opportunities and solutions

The guided conversations highlighted three areas of concern: 1) specific patient needs; 2) delivering proactive care; and 3) supporting patients to self-manage.

***Specific patient needs.*** Patient’s responses to the pandemic ranged widely. While some appeared to adapt to the new service delivery model, others struggled when face-to-face contacts were decreased or replaced with telehealth. The main concerns centred on communication challenges and service contractions.

***Communication challenges.*** Some clients either did not have access to telephones or smartphones with data. Others, for a variety of reasons, were reluctant to take calls from restricted numbers. Solutions had to be tailored to the specific needs and circumstances of individuals. Those without phones were often also more socially marginalised and isolated, with limited to no natural support networks. Reach out to this group was increased and they were encouraged to stay in touch with services. Information on how telehealth could help to keep them safe was also provided. Many were supported to purchase inexpensive phones using their welfare benefits. For those reluctant to receive phone calls without caller identification, arrangements were made for a text to be sent alerting them of an incoming the phone call.

Clients who have had an adversarial relationship with services which may, knowingly or unknowingly, conceal signs of deterioration were of particular concern as telehealth could be inadequate to identify changes in mental state. For this group, when possible, family and/or friends were contacted and provided with support and information to identify early warning signs. When patients did not have a support network available, they continued to be seen face to face.

Communication issues via telehealth for those with limited English or sensory deficits such as deafness were resolved using three-way telehealth sessions, involving foreign language interpreters or sign language interpreters for those with deafness. For this to occur, patients needed to have access to smartphones with data and video capability.

***Service contraction challenges.*** The impact of the reduction of other services (i.e. general practice, phlebotomy, pharmacies and pathology) was also considered. Many patients have physical comorbidities that are managed through their general practitioner or are under shared care arrangements. The decrease in phlebotomy and pathology services affected patients taking clozapine. Changes in the ways that pharmacies delivered services affected the patients taking regular medication or daily methadone. To comply with physical distancing and due to limited access to personal and protective equipment, many non-government organisations (NGOs) reduced their services. This affected homecare services such as helping clients to maintain personal hygiene, daily meals, medication intake, procure essential supplies or carry out weekly shopping. Patients who would be affected by any of these contractions were identified and alternate individualised plans were devised to ensure continuity of care.

***Proactive care.*** Case managers were also supported to think through, at an individual patient level, how they might continue to deliver care. Patients were proactively contacted through telehealth at a minimum of once a week. These frequent contacts were comparatively shorter and were aimed to deliver focussed interventions. The contacts needed to be assets focussed, drawing on the person’s strengths and natural circle of support to self-manage. The aim of these focussed interventions was to support self-management as opposed to patients feeling deserted and having to self-manage to survive.

***Case managers’ concerns.*** Many case managers raised concerns that weekly contacts could unnecessarily bring to the surface crises faced by patients diagnosed with borderline personality disorder, which the patient would have otherwise dealt with on their own. Others were concerned that if they were to frequently contact patients diagnosed with chronic paranoia and schizophrenia, the patients would resist and intrusion from the contacts could potentially worsen their presentation.

***Self-management.*** To address the proactive care concerns, case managers were encouraged to explicitly state to patients that the focus of the weekly contacts was on supporting self-management. Simple conversation scripts around how patients can maintain their well-being and access essential supplies for their day-to-day living were distributed (Table 2). These scripts enabled the case manager to connect on a human level, without the patients feeling checked on.

Some case managers were concerned about patients who, as part of their treatment, had weekly face-to-face formal therapy. Case managers were encouraged to explain to patients why their therapy was interrupted and how service continuity would be maintained through the crisis. It was also reinforced that it was important to keep patients up to date as to when formal therapy would be reinitiated. Through team discussion, patients for whom these sessions needed to continue were identified.

## Discussion

The guided conversations provided the opportunity to think outside the confines of conventional care and to refocus the care towards the assets and strengths of the patient. The approach also allowed case managers to prepare patients for staffing shortfalls by encouraging them to draw on their support network and engage in meaningful activities which they could continue during lock down. Keeping with recovery philosophy, the focus of self-management was on maintaining a meaningful life and not just mitigation of risk. This dual focus was central to staying safe and keeping well.

### Limitations of the synopsis

The purpose of the synopsis was to support triage and prioritisation of patients’ needs, in the eventuality of extensive workforce reduction. There were, however, inconsistencies around the information provided. Most case managers were concerned that the short format would result in key information being missed. The conversational crosscheck with the consultants picked up critical risk- or safety-related information that was absent or was not synthesised into what it meant in terms of mode and frequency of contact. Forward planning, as to what would make the situation unsafe, was also absent in many and needed inclusion.

## Conclusion

Mental health services aspire to deliver person-centred care. However, during COVID-19, the focus had to shift from the person to the community. Keeping the community safe through staying at home, reducing travel and decreasing face-to-face contact could have come at the price of the person being unsupported and becoming unwell or unsafe. The contingency planning attempted to address these concerns through thoughtful consideration of the strengths and needs of each individual patient. The staffing implications of the pandemic forced professionals to consider recovery-oriented practice of supported self-management. For this, staff had to move from ‘top to tap,’^1^ a shift in thinking from a deficit-oriented directive approach of ‘what’s the matter with you’ that I will fix to an assets-based approach of ‘what matters to you’ that I can support in partnership with you.^2^ The act of summarising a life into 90 words and consequent discussions highlighted that what matters to people living with mental health challenges is no different to those without. We all want a safe and meaningful life, productive livelihood, supportive relationships and contributions to society. Albeit born from a crisis, the contingency planning for COVID-19 highlighted the role of self-management and the need to focus on assets rather than deficits. There was also the shared experience of threat to life and livelihood, and neither the patient nor staff was immune to the parallel pandemic of psychological distress. Perhaps, COVID-19 will dent the power differential between professionals and patients, allowing us to connect at a human level and continue to remind us in healthcare that ‘what matters to you matters to me too’.
